# The risk of acute thromboembolic cardiovascular events in association with use of chondroitin sulphate: A comparative, propensity score-matched cohort study using Swiss healthcare claims data

**DOI:** 10.1016/j.ocarto.2026.100791

**Published:** 2026-04-02

**Authors:** Tamino Zappalà, Theresa Burkard, Carola A. Huber, Christoph R. Meier, Julia Spoendlin

**Affiliations:** a Basel Pharmacoepidemiology Unit, Division of Clinical Pharmacy and Epidemiology, Department of Pharmaceutical Sciences, University of Basel, Basel, Switzerland; b Nuffield Department of Orthopaedics, Rheumatology and Musculoskeletal Sciences, University of Oxford, Oxford, UK; c Department of Health Sciences, Helsana Group, Zurich, Switzerland; d Hospital Pharmacy, University Hospital Basel, Basel, Switzerland

**Keywords:** Chondroitin sulphate, Cardiovascular events, Drug safety study, Healthcare claims data

## Abstract

**Objective:**

We aimed to evaluate the risk of acute thromboembolic cardiovascular events in new chondroitin (sulphate) users compared to new users of intraarticular (IA) steroids in a propensity score (PS)-matched cohort study.

**Method:**

Using Swiss healthcare claims data (2013–2022, study period), we identified new users of chondroitin and IA steroids aged 40–99 years. Primary outcome was an inpatient-diagnosed myocardial infarction, ischemic stroke, or transient ischemic attack. The secondary outcome additionally included all-cause mortality. After PS matching, we calculated incidence rates (IRs) and estimated hazard ratios (HRs) with 95% confidence intervals (CIs). In a sensitivity analysis, we restricted to patients with imaging of the hip/knee and a claim for NSAIDs within 365 days before cohort entry to increase likelihood of them having osteoarthritis (OA).

**Results:**

After matching, we included 36,659 chondroitin users and 37,501 IA steroid users. Matched IRs for the primary outcome were 9.6 for chondroitin and 11.9/1000 py for IA steroids, yielding a matched (m)HR of 0.81 (95% CI 0.69–0.95). The sensitivity analysis yielded a mHR of 0.79 (95% CI 0.56–1.11). IRs for the secondary outcome were 18.9 and 29.2/1000 py for chondroitin and IA steroid users, resulting in a mHR of 0.67 (95% CI 0.60–0.75).

**Conclusion:**

Chondroitin use was associated with a slightly lower risk of cardiovascular events compared to IA steroid use. However, residual confounding by patient frailty may explain the finding to some degree, given the excess mortality in the comparison group. Further research is needed to corroborate or refute a potential cardioprotective effect of chondroitin.

## 1 Introduction

Chondroitin sulphate (referred to as ‘chondroitin’) modifies osteoarthritis (OA) symptoms by inhibiting the cartilage-degrading elastase, thereby increasing the water binding capacity of the cartilage [1]. In Switzerland, chondroitin is indicated and commonly prescribed for OA and reimbursed by mandatory basic health insurance.

Prior case-control studies based on Spanish primary care data reported a 43% and 23% reduced relative risk of myocardial infarction (MI) and ischemic stroke in association with new chondroitin use when compared to non-use [2,3]. This risk reduction was even stronger in subgroups with cardiovascular risk factors [2,3]. The study also evaluated glucosamine use, which was neutral in association with myocardial infarction [2], but also associated with a reduced relative risk of ischemic stroke [3]. In Switzerland, glucosamine is only available in nutritional supplements and is not reimbursed by mandatory basic health insurance. The Spanish authors adjusted for covariates body-mass-index (BMI), smoking, alcohol use, specific cardiovascular comorbidities and comedications, but not for the presence of an osteoarthritis diagnosis (i.e. the indication of OA). Both studies compared chondroitin use to non-use, which introduces the risk of residual confounding by indication or by patient frailty [4].

Nevertheless, the observed reduced risk of cardiovascular events in association with chondroitin is intriguing, given that numerous epidemiological studies previously reported an increased cardiovascular risk in patients with OA when compared to the general population [[5], [6], [7], [8]]. This cardiovascular risk increased with increasing severity of OA [9,10]. This may be the result of chronic low-grade inflammation, the lack of physical activity, or a combination of both.

We aimed to evaluate the risk of acute thromboembolic cardiovascular events in association with new use of chondroitin compared to new users of IA steroid injections, which are also indicated for the treatment of OA, in a propensity score (PS)-matched cohort study using Swiss healthcare claims data.

## 2 Methods

### 2.1 Study design & data source

We performed a retrospective, PS-matched new user cohort study using claims data from the Helsana Group between January 1, 2012, and December 31, 2022. Of 45 different health insurance companies in 2022, the Helsana Group is one of the largest in Switzerland, covering about 16% of the Swiss population with basic mandatory health insurance (approx. 1.4 million individuals in 2022) from all 26 cantons [11]. The Helsana claims database provides anonymized information on demographics, insurance model, start and end of insurance coverage, outpatient procedures and consultations (TARMED codes), bundled diagnostic information on hospitalisations (SwissDRG codes), and all drug claims (ATC codes), including information on the dispensation, strength, formulation and pack size, as well as information on prescriber and service provider.

### 2.2 Exposure definition

We identified all new users of chondroitin (ATC M01AX25, only 400 mg and 800 mg products, not 500 mg, because the recommended dose is 800 mg per day [1]) aged 40–99 years between 2013 and 2022 (further referred to as chondroitin users). New use was defined as the first claim of a study drug after at least one year of unexposed history in the database. The date of this first claim was defined as the cohort entry date (CED). We excluded patients with either a recorded SwissDRG code for an outcome of interest (defined below) at any time before CED, or a medication claim for an active comparator within 365 days before the CED. The CED of each patient was then allocated to one of ten 1-year cohort entry blocks throughout the study period. Using the strength and package size of the claimed chondroitin product, we calculated the duration of supply with chondroitin per claim according to standard dosing (800 mg per day).

### 2.3 Comparison group

We compared chondroitin users to patients who received IA steroid injections (from here on referred to as IA steroid users). Exposure to IA steroid injections was defined as a combination of a recorded procedure code for an intraarticular injection (TARMED 24.0130) and a recorded claim for an injectable corticosteroid (i.e. triamcinolone, Supplementary Table S1) on the same date. We identified all patients who received IA steroid injections aged 40–99 years between 2013 and 2022. The CED was the first IA steroid injection after at least one year without exposure. IA steroid injections can be given up to four times per year, but most patients received them less frequently, and we therefore considered patients exposed for 365 days after each injection with an additional 90-days grace period.

### 2.4 Follow-up and censoring

Patients could enter the cohort both as exposed or unexposed if inclusion criteria were met (though not at the same time). We followed chondroitin users from the CED for as long as they were exposed until the outcome of interest occurred or they were censored when (1) the study period ended (December 31, 2022), (2) insurance coverage ended, (3) the patient died, (4) the patient had a recorded claim for a comparator drug, (5) 90 days after (grace period) the patient stopped using chondroitin (i.e. supply ended without a subsequent claim during the grace period), or they (6) reached 3 years of follow-up. For IA steroid users, we allowed a follow-up of one year after each injection and censored on day 456 (365 days plus 90-day grace period) if not followed by a subsequent injection. All other censoring criteria were identical.

### 2.5 Outcome definition

The primary outcome of interest was an inpatient-diagnosed fatal or non-fatal acute thromboembolic event, including MI or ischemic stroke/transient ischemic attack, identified using SwissDRG codes (Supplementary Table S2).

The secondary outcome additionally included all-cause mortality to capture patients with a fatal MI or stroke who died before hospital admission (not captured in SwissDRG) and to compare overall patient frailty (cause of death data not captured).

To evaluate the potential role of residual confounding by BMI in our analyses, we analysed ‘surgery for a hernia’ as a negative control outcome (SwissDRG codes, Supplementary Table S3). The occurrence of hernias is strongly correlated with higher BMI [12], a variable which is also associated with OA but is not captured in Swiss claims data.

### 2.6 Statistical analysis

To control for confounding we conducted PS matching. Therefore, we estimated a PS for each patient, using multivariable logistic regression [13], including variables recorded prior to the CED and selected *a priori* based on clinical knowledge constituting potential confounders or predictors of the outcome (Table 1). We then created a comparative cohort (chondroitin vs. IA steroids), matching a maximum of two comparator patients to each chondroitin user on their PS. We used a greedy matching algorithm, which matched up to the eighth digit of the PS to the nearest neighbour, excluding those who failed to match. Matching was performed sequentially within each of the ten 1-year entry blocks (2013–2022) to account for potentially different healthcare or drug use over time.

**Table 1:** Distribution of baseline covariates of chondroitin users and IA steroid users before and after PS matching.

	Before PS-matching			PS-matched		
	Chondroitin (N = 90 060)	IA steroids (N = 37 726)	SMD	Chondroitin (N = 36 659)	IA steroids (N = 37 501)	SMD
Median age [years] (IQR)	63 (54–73)	66 (55–76)	−0.173	66 (55–76)	66 (55–76)	−0.032
Median follow-up [years] (IQR)	0.5 (0.5–0.7)	1.2 (1.2–1.2)	N/A	0.5 (0.5–0.7)	1.2 (1.2–1.2)	N/A
Female	58 616 (65.1%)	22 346 (59.2%)	−0.121	17 948 (58.0%)	18 481 (58.6%)	0.019
Male	31 444 (34.9%)	15 380 (40.8%)	−0.121	12 979 (42.0%)	13 063 (41.4%)	0.019
Median number of hospital contacts 365d before CED (IQR)	0 (0–0)	0 (0–0)	−0.160	0 (0–0)	0 (0–0)	−0.031
Median number of claims (of medications listed below) 365d before CED (IQR)	6 (3–11)	8 (5–14)	−0.278	8 (4–14)	8 (5–14)	−0.038
**Medications 365d prior CED:**						
Reflux medication (ATC A02)	40 288 (44.7%)	20 309 (53.8%)	−0.121	19 436 (53.0%)	20 106 (53.6%)	−0.012
Antidiabetics (ATC A10)	8132 (9.0%)	4177 (11.1%)	−0.183	4004 (10.9%)	4128 (11.0%)	−0.003
Anticoagulants (ATC B01AA/B01AB/B01AF)	11 463 (12.7%)	6098 (16.2%)	−0.068	5765 (15.7%)	6012 (16.0%)	−0.008
Blood substitutes (ATC B05)	21 287 (23.6%)	10 963 (29.1%)	−0.098	10 373 (28.3%)	10 834 (28.9%)	−0.013
Cardiac therapy (ATC C01)	8727 (9.7%)	4473 (11.9%)	−0.123	4230 (11.5%)	4429 (11.8%)	−0.008
Diuretics (ATC C03)	7574 (8.4%)	4770 (12.6%)	−0.138	4333 (11.8%)	4674 (12.5%)	−0.020
Vasodilators (ATC C04)	11 611 (12.9%)	4913 (13%)	−0.004	4658 (12.7%)	4882 (13.0%)	−0.009
Betablockers (ATC C07)	16 123 (17.9%)	8199 (21.7%)	−0.096	7638 (20.8%)	8109 (21.6%)	−0.019
Calcium-channel blockers (ATC C08)	8788 (9.8%)	4716 (12.5%)	−0.087	4341 (11.8%)	4653 (12.4%)	−0.017
RAAS-blockers (ATC C09)	29 633 (32.9%)	14 449 (38.3%)	−0.113	13 725 (37.4%)	14 315 (38.2%)	−0.015
Lipid-modifiers (ATC C10)	21 294 (23.6%)	10 156 (26.9%)	−0.075	9627 (26.3%)	10 071 (26.9%)	−0.013
Secondary antihypertensives (ATC C02)	625 (0.7%)	362 (1.0%)	−0.029	347 (1.0%)	356 (1.0%)	0.00
Thyroid therapy (ATC H03)	7597 (8.4%)	3195 (8.5%)	−0.001	3054 (8.3%)	3176 (8.5%)	−0.005
Anti-inflammatory and antirheumatic products (ATC M01A, excl. Chondroitin sulphate)	58 420 (64.9%)	26 907 (71.3%)	−0.139	26 058 (71.1%)	26 725 (71.3%)	−0.004
Topical pain medication (ATC M02)	35 196 (39.1%)	14 999 (39.8%)	−0.014	14 213 (38.8%)	14 890 (39.7%)	−0.019
Opioids (ATC N02A)	13 405 (14.9%)	8654 (22.9%)	−0.207	7978 (21.8%)	8485 (22.6%)	−0.021
Analgesics (ATC N02B)	41 316 (45.9%)	20 136 (53.4%)	−0.150	19 094 (52.1%)	19 939 (53.2%)	−0.022
Psycholeptics (ATC N05)	23 448 (26.0%)	10 876 (28.8%)	−0.063	10 215 (27.9%)	10 765 (28.7%)	−0.019
Bone drugs (ATC M05B)	3977 (4.4%)	2208 (5.9%)	−0.065	2005 (5.5%)	2174 (5.8%)	−0.014
**Procedures 365d before CED**						
Outpatient arthroscopy	1183 (1.3%)	617 (1.6%)	−0.027	633 (1.7%)	613 (1.6%)	0.007
MRI	16 885 (18.8%)	10 730 (28.4%)	−0.230	10 165 (27.7%)	10 608 (28.3%)	−0.012
X-ray	30 897 (34.3%)	12 587 (33.4%)	0.020	12 192 (33.3%)	12 521 (33.4%)	−0.003
Arthroplasty (any time before CED)	849 (0.9%)	776 (2.1%)	−0.092	598 (1.6%)	742 (2.0%)	−0.026
**Cohort entry blocks**						
2013	11 534 (12.8%)	4678 (12.4%)	N/A	4558 (12.4%)	4662 (12.4%)	N/A
2014	10 050 (11.2%)	4304 (11.4%)	N/A	4209 (11.5%)	4275 (11.4%)	N/A
2015	8958 (9.9%)	3924 (10.4%)	N/A	3794 (10.4%)	3897 (10.4%)	N/A
2016	8824 (9.8%)	4051 (10.7%)	N/A	3963 (10.8%)	4043 (10.8%)	N/A
2017	8696 (9.7%)	3536 (9.4%)	N/A	3482 (9.5%)	3515 (9.4%)	N/A
2018	8271 (9.2%)	3391 (9.0%)	N/A	3297 (9.0%)	3373 (9.0%)	N/A
2019	8465 (9.4%)	3562 (9.4%)	N/A	3417 (9.3%)	3542 (9.5%)	N/A
2020	7686 (8.5%)	3294 (8.7%)	N/A	3188 (8.7%)	3260 (8.7%)	N/A
2021	8572 (9.5%)	3369 (8.9%)	N/A	3244 (8.9%)	3340 (8.9%)	N/A
2022	9004 (10.0%)	3617 (9.6%)	N/A	3507 (9.6%)	3594 (9.6%)	N/A

We compared the distribution of all baseline covariates before and after matching (Table 1 and Table S4). We calculated crude and matched incidence rates (IRs) per 1000 person-years (py) for the primary and secondary outcomes. Using Cox proportional hazard regression analyses, we calculated hazard ratios (HRs) with 95% confidence intervals (CI, using a robust variance estimator) for the relative risk of acute thromboembolic cardiovascular events in association with use of chondroitin versus IA steroids. We calculated an overall HR in all chondroitin users versus IA steroids, as well as time-specific HRs within the first, second, and third year as a proxy for the cumulative dose, excluding patients whose follow-up ended prior to the time-period of interest. In addition to PS-matched HRs, we present crude and multivariable adjusted (all covariates that were included in the PS) HRs for all analyses.

The same types of analyses were conducted to quantify HRs for the secondary outcome including all-cause mortality and for the negative control outcome ‘hernia surgery’.

Because we lost a large proportion of exposed patients during PS-matching, we post-hoc also applied PS fine stratification on the PS for the main analysis of our primary outcome. This method allows to retain almost all patients in both comparison groups [14].

### 2.7 Subgroup, sensitivity and additional analyses

We performed subgroup analyses, by sex and by age (40–69 and 70–99 years), for which we re-matched patients within subgroups. We also performed five sensitivity analyses with stricter inclusion criteria to increase comparability of the comparison groups. For those, we only included (1) patients without cardiovascular risk factors, defined as not having any claims for medications associated with cardiovascular risk (i.e. antidiabetics, diuretics, betablockers, calcium-channel blockers, renin-angiotensin-aldosterone system (RAAS)-blockers, secondary antihypertensives, lipid-modifiers, anti-obesity medication, or cardiac therapy) within 365 days before CED, (2) patients with cardiovascular risk factors, defined as having one of the above mentioned medications associated with cardiovascular risk 365 days before CED, (3) patients who had undergone an MRI and/or X-ray of the hip and/or knee 365 days before CED to increase the likelihood of them having OA (of the same location), (4) patients having undergone an MRI and/or X-ray of the hip and/or knee as well as a claim for nonsteroidal anti-inflammatory drugs within 365 days before CED to further increase the likelihood of a present OA diagnosis. This sensitivity analysis was also repeated separately for women, because women revealed a strong overall HR in the primary analysis. Finally, (5) patients who had a claim for a reflux medication (mainly proton-pump inhibitors) within 365 days before CED as an indicator for patient frailty.

To evaluate robustness of our exposure definition, we performed an overall as-started analysis, where we followed patients in their group for a maximum of 3 years regardless of exposure changes or discontinuation (similar to an intention-to-treat approach in a randomised controlled trial, RCT), until they had an outcome or were censored due to defined censoring criteria. We also performed an as-started analysis restricted to patients with one single claim for chondroitin before supply ended. For this analysis, we implemented a run-in period of 180 days and started follow-up on day 181 after the CED (excluding patients whose follow-up ended before). This analysis was implemented to evaluate potential confounding by indication, because any association that is observed when comparing patients after they have stopped taking the drug of interest is more likely caused by underlying confounding rather than by a causal effect (assuming that a single claim of chondroitin does not sustainably reduce the cardiovascular risk).

## 3 Results

**Fig. 1:**
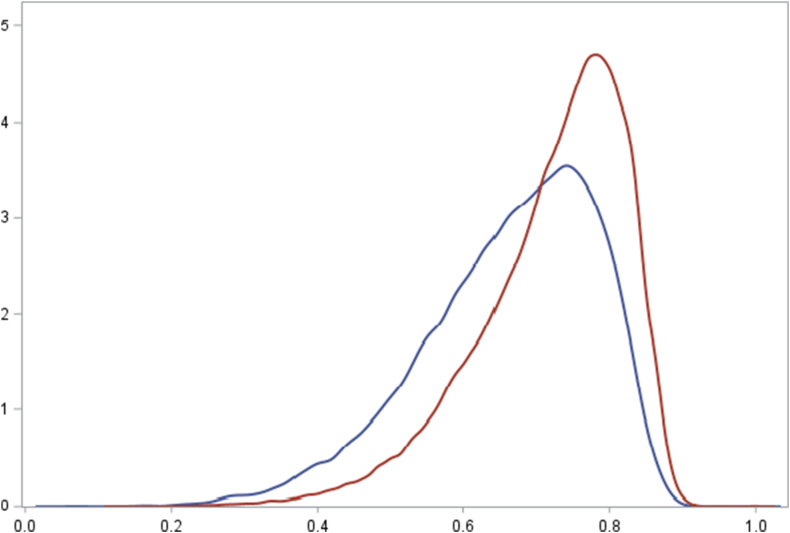
Distribution of the PS in chondroitin users (red) and new IA steroid users (blue) before PS matching. (For interpretation of the references to color in this figure legend, the reader is referred to the Web version of this article.)

After PS-matching, we included 36,659 chondroitin users matched to 37,501 IA steroid users (90,060 chondroitin and 37,726 IA steroid users before matching), of whom 3397 were included in both comparison groups (1197 first in the chondroitin group and 1200 first in the IA steroid group). The PS distribution for chondroitin and IA steroid users had sufficient overlap before matching (Fig. 1). After matching, all covariates had a standardized mean difference of <0.1. (Table 1). Due to the smaller size of the IA steroid group, the comparator group limited the matching; 99.4% of IA steroid users were retained in the PS-matched cohort vs. 40.7% of chondroitin users. Before matching, IA steroid users were slightly older and less likely female, and also slightly more often claimed certain comedications (Table 1). The median follow-up was 0.5 years (IQR 0.5–0.7) for chondroitin users and 1.2 years (IQR 1.2–1.2) for IA steroid users. The median follow-up after matching remained the same. Median number of follow-up days of chondroitin users was 180 (IQR 180–270).

### 3.1 Cox proportional hazard estimation

In our matched primary analysis, we captured a total of 793 thromboembolic cardiovascular events during follow-up (257 [0.7%] in chondroitin users and 426 [1.4%] in IA steroid users). After matching, we observed slightly lower IRs for cardiovascular events among chondroitin users (9.6/1000 py) compared to IA steroid users (11.9/1000 py), yielding a matched (m)HR of 0.81 (95% CI 0.69–0.95). The crude HR was 0.59 (95% CI 0.52–0.68), and the multivariable adjusted HR 0.77 (95% CI 0.67–0.88, Table 2).

**Table 2:** Results of the association between chondroitin (vs. intraarticular steroids) and acute thromboembolic cardiovascular events before and after PS-matching overall and in subgroups, incl. sensitivity analyses.

	Before PS-matching				PS-matched		
	Obs.-time^a^ in chondroitin + IA steroid users	Events in chondroitin + IA steroid	HR crude (95% CI)	HR adjusted^b^ (95% CI)	Obs.-time^a^ in chondroitin + IA steroid users	Events in chondroitin + IA steroid	HR matched^b^ (95% CI)
**Primary outcome**							
Overall	64 533 + 45 265	449 + 546	0.59 (0.52–0.68)	0.77 (0.67–0.88)	26 831 + 45 001	257 + 536	0.81 (0.69–0.95)
***Duration of follow-up***							
1st year	52 411 + 33 491	334 + 401	0.55 (0.47–0.64)	0.73 (0.63–0.85)	21 452 + 33 298	190 + 397	0.76 (0.63–0.91)
2nd year	8284 + 10 604	76 + 127	0.80 (0.59–1.08)	0.89 (0.65–1.20)	3639 + 10 541	40 + 123	0.99 (0.68–1.45)
3rd year	3838 + 1170	39 + 18	0.66 (0.38–1.16)	0.88 (0.49–1.57)	1740 + 1162	27 + 16	1.08 (0.58–2.03)
**Sex**							
Women	40 545 + 19 987	268 + 341	0.51 (0.43–0.61)	0.75 (0.63–0.89)	16 078 + 26 686	157 + 329	0.79 (0.64–0.97)
Men	22 211 + 18 313	181 + 205	0.77 (0.62–0.95)	0.83 (0.67–1.02)	10 495 + 18 029	102 + 199	0.94 (0.73–1.22)
**Age in years**							
40–69	40 825 + 26 110	93 + 82	0.81 (0.59–1.11)	0.89 (0.65–1.24)	14 792 + 25 906	39 + 82	0.91 (0.59–1.39)
70–99	23 708 + 19 155	356 + 464	0.62 (0.54–0.72)	0.76 (0.65–0.88)	11 762 + 18 873	214 + 455	0.76 (0.64–0.90)
**Secondary outcome**^c^							
Overall	65 502 + 47 124	886 + 1383	0.49 (0.44–0.53)	0.66 (0.61–0.73)	27 905 + 45 928	528 + 1343	0.67 (0.60–0.75)
***Duration of follow-up***							
1st year	52 873 + 34 916	636 + 1010	0.44 (0.40–0.49)	0.64 (0.57–0.71)	22 316 + 33 997	373 + 978	0.61 (0.54–0.70)
2nd year	8564 + 10 913	166 + 322	0.67 (0.55–0.82)	0.76 (0.62–0.92)	3783 + 10 705	101 + 316	0.93 (0.73–1.18)
3rd year	4065 + 1292	82 + 51	0.51 (0.36–0.72)	0.73 (0.51–1.06)	1806 + 1227	53 + 49	0.71 (0.47–1.08)
**Sensitivity analyses**							
Without CV risk^d^	26 976 + 17 446	81 + 82	0.68 (0.49–0.94)	0.76 (0.55–1.07)	9511 + 17 249	34 + 81	0.78 (0.50–1.21)
With CV risk^d^	37 556 + 27 819	368 + 464	0.60 (0.52–0.69)	0.77 (0.66–0.89)	17 075 + 27 604	223 + 460	0.79 (0.67–0.93)
With MRI/X-ray^e^	24 723 + 15 161	165 + 178	0.60 (0.48–0.75)	0.89 (0.71–1.12)	9236 + 14 917	87 + 170	0.86 (0.66–1.13)
With MRI/X-ray^e^ + NSAID	17 945 + 11 545	109 + 121	0.62 (0.47–0.81)	0.88 (0.67–1.16)	6902 + 11 339	54 + 117	0.79 (0.56–1.11)
With MRI/X-ray^e^ + NSAID (only women)	11 560 + 7359	70 + 82	0.59 (0.42–0.83)	0.92 (0.66–1.30)	4398 + 7157	40 + 78	0.87 (0.59–1.30)
With reflux drugs	29 034 + 24 462	262 + 335	0.69 (0.58–0.82)	0.86 (0.72–1.02)	14 260 + 24 181	157 + 323	0.84 (0.68–1.02)
**As-started analysis**							
Overall	226 399 + 93 659	1520 + 1032	0.61 (0.56–0.66)	0.83 (0.77–0.90)	92 146 + 93 207	826 + 1018	0.82 (0.75–0.90)
***Duration of follow-up***							
1st year	84 989 + 35 443	543 + 413	0.55 (0.48–0.62)	0.75 (0.66–0.86)	34 585 + 35 243	289 + 409	0.72 (0.62–0.84)
2nd year	75 289 + 31 136	512 + 338	0.63 (0.55–0.72)	0.85 (0.74–0.98)	30 689 + 30 986	290 + 333	0.88 (0.75–1.03)
3rd year	66 121 + 27 080	462 + 281	0.88 (0.75–1.03)	0.68 (0.58–0.79)	26 872 + 26 978	247 + 276	0.90 (0.76–1.07)
**Patients with 1 rx**^f^							
Overall	143 234 + 92 879	943 + 1049	0.58 (0.53–0.64)	0.87 (0.80–0.96)	80 059 + 89 846	680 + 958	0.80 (0.72–0.88)
**Negative control outcome**							
Hernia surgery	71 853 + 48 266	302 + 220	0.91 (0.76–1.10)	1.02 (0.84–1.23)	30 897 + 46 761	170 + 214	1.17 (0.94–1.46)
**Primary outcome – PS fine stratification**							
Overall	64 533 + 45 265	449 + 546	0.59 (0.52–0.68)	N/A	64 530 + 45 180	449 + 395	0.83 (0.72–0.95)

a Observation-time in person-years after exposure.

b Adjusted for/PS estimation with covariates (from Table 1).

c Acute thromboembolic cardiovascular event or all-cause mortality.

d At least one claim within 365 days before exposure to chondroitin sulphate for following medications: anti-obesity, antidiabetics, cardiac medications, antihypertensive, diuretics, betablockers, calcium-channel blockers, RAAS-blockers, lipid-modifiers.

e Including only patients with MRI and/or X-ray for hip and/or knee within 365 days before CED.

f Only including patients who had a single claim for chondroitin, applying a 180-day run in period.

**Fig. 2:**
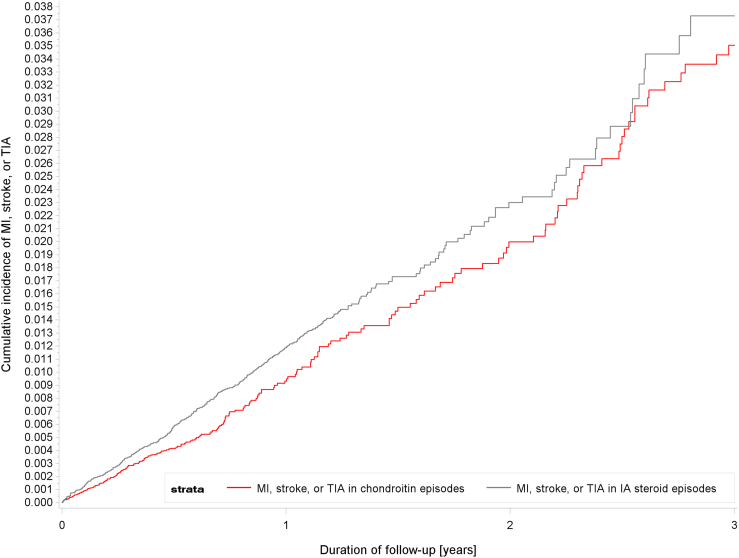
Cumulative incidence of MI, stroke, or transient ischemic attack over the duration of follow-up in chondroitin users and IA steroid users.

When quantifying time-specific HRs, we observed a strong mHR for thromboembolic cardiovascular events of 0.76 (95% CI 0.63–0.91) during the first year of follow-up, which became a null result in the second and third year of follow-up (mHR 0.99; 95% CI 0.68–1.45/mHR 1.08; 0.58–2.03), but sample size in the second and third year of follow-up was small (206 events in total). Fig. 2 shows the cumulative incidence of outcome events during follow-up.

For the secondary composite outcome of acute thromboembolic cardiovascular events or all-cause mortality, we observed matched IRs of 18.9 per 1000 py among chondroitin users and 29.2 per 1000 py among IA steroid users. We observed an overall mHR of 0.67 (95% CI 0.60–0.75), which was stronger during the first year of follow-up (mHR 0.61: 95% CI 0.54–0.70) but was not significant in the second year of follow-up (mHR 0.93; 95% CI 0.73–1.18, Table 2).

Stratification of our primary outcome analysis by the presence of cardiovascular risk factors did not meaningfully change results but the number of outcome events was small in patients without risk factors (115 events). Restricting to persons with hip and/or knee MRI and/or X-ray and at least one claim of an NSAID yielded a mHR of 0.79 (95% CI 0.56–1.11, Table 2). Subgroup analyses revealed a stronger effect estimate among women (mHR 0.79; 95% CI 0.64–0.97) than in men (mHR 0.94; 95% CI 0.73–1.22). The mHR was 0.87 (95% CI 0.59–1.30) for women with prior imaging and at least one claim for an NSAID. Patients over 70 years of age revealed a mHR of 0.76 (95% CI 0.64–0.90), those under 70 years of age a mHR of 0.82 (95% CI 0.75–0.90), which was stronger during the first year of follow-up (mHR 0.72; 95%CI 0.62–0.84) and became statistically non-significant with longer duration of follow-up Patients with only one claim of chondroitin during follow-up yielded an overall mHR of 0.80 (95% CI 0.72–0.88, Table 2).

In the PS fine stratification analysis, >99.9% of all patients were retained in both groups. Results were comparable to the overall PS-matched analysis with a HR of 0.83 (95% CI 0.72–0.95, Table 2).

### 3.2 Negative control outcomes

After matching, we observed no significant association with the negative control outcome hernia surgery (mHR 1.17; 95% CI 0.94–1.46) when comparing chondroitin users to IA steroid users (Table 2).

## 4 Discussion

In this comparative, PS-matched cohort study using Swiss claims data, we observed a slightly reduced risk of thromboembolic cardiovascular events when comparing new chondroitin users to new IA steroid users. However, we also observed higher all-cause mortality in the comparison group, suggesting some potential for residual confounding by unmeasured patient frailty.

Two Spanish case-control studies previously reported reduced relative risks of thromboembolic cardiovascular events using primary-care data [2,3]. They observed an overall adjusted odds ratio for MI of 0.57 (95% CI 0.46–0.72) [2] and for ischemic stroke of 0.77 (95% CI 0.60–0.99), when comparing ongoing new use of chondroitin to non-use, whereas past use of chondroitin was not associated [3]. In contrast to our study, they had information on BMI for which they adjusted. However, although they had access to outpatient diagnoses, they did not control for diagnoses of OA, which is the indication for chondroitin and which has been associated with an increased risk of cardiovascular events [[5], [6], [7]]. As opposed to chondroitin users, the majority of non-users likely did not have OA, thus introducing confounding by indication. Despite them controlling for healthcare utilisation, it is possible that non-users had a different cardiovascular risk profile. We performed a PS-matched cohort study, using an active comparator group (IA steroid injections). PS matching helps to balance covariates between chondroitin users and comparators and thus mitigates measured confounding. We also observed overall strong HRs for fatal or non-fatal acute MI or ischemic stroke/transient ischemic attack in our data, but effect size was smaller than those reported in the Spanish study (19% [combined outcome in our study] vs. 43% [myocardial infarction] and 23% [ischemic stroke] risk reduction in prior studies).

The choice of the comparator group is a key aspect in comparative observational studies. Ideally, the comparator group shares the same indication and initiates a drug treatment for this same indication of the same severity at the same time as the exposed group, akin to an RCT [4]. However, such a comparator group is not always available. In Switzerland, the outpatient health care system is based on a fee-for-service billing system and does not record outpatient diagnoses, such as OA. Given that chondroitin is exclusively indicated for OA, we assumed that all exposed patients have OA of any joint. To increase the likelihood of the comparator group also having OA, we chose IA steroid injections as the comparator, because IA steroids are also indicated for the management of OA (mainly knee OA), besides other indications such as psoriatic arthritis or gout [1,15]. However, the lack of OA diagnoses in our database is one of the main limitations of our study. Furthermore, not all OA locations may share the same cardiovascular risk. Knee OA has greater potential to limit physical activity than OA in other locations (e.g. hands), and may thus increase the cardiovascular risk per se. Although knee and hip OA is most common [16], the lack of adjustment by OA location might involve potential residual confounding. To further increase the likelihood of the comparator patients to have OA of similar location, we performed sensitivity analyses only including patients who had an MRI and/or X-ray of the hip or knee as well as a claim for an NSAID within 365 days before CED. For our primary comparison, mHRs remained below one despite this restriction, suggesting that confounding by OA-location is not the main driver of our observed results (mHR 0.79; 95% CI 0.56–1.11).

Subgroup analyses revealed a stronger risk reduction among women than men, but the sensitivity analysis including only women who had an MRI and/or X-ray of the hip or knee as well as a claim for an NSAID within 365 days before CED, yielded a mHR closer to the null, which indicates potential residual confounding among women, in whom alternative indications for IA steroids such as psoriasis or rheumatoid arthritis are more common. The sensitivity analysis including patients with reflux medication did not meaningfully change the effect estimate. Subgroup analyses by age revealed a stronger effect estimate in patients aged 70 years or older, but the number of outcome events was small in younger patients.

We evaluated hernia surgery as a negative control outcome to assess for potential residual confounding by obesity, because obesity is a risk factor for developing hernia as well as OA. This analysis revealed no association, suggesting no residual confounding by obesity in our analysis.

We quantified IRs for acute thromboembolic cardiovascular events of 9.6 per 1000 py among chondroitin users and 11.9 per 1000 py among IA steroid users, which is lower than the combined incidence of MI and ischemic stroke in the Swiss population aged 50–99 years (18/1000 citizens) [17,18]. It is possible that patients receiving ‘preventive’ treatments such as chondroitin are overall healthier than the Swiss population of the same age. However, we also missed those MIs and ischemic strokes, which resulted in death outside the hospital or after admission to the emergency room without an inpatient stay. To include these potentially missed events, we analysed a secondary composite outcome, which additionally included all-cause mortality (the database does not capture cause-of-death data). We observed an IR of 18.9 per 1000 py for chondroitin users and a higher IR of 29.2 per 1000 py for IA steroid users. While the IR of the secondary composite outcome for chondroitin only slightly exceeds the overall number of deaths by cardiovascular diseases in Switzerland per year (approx. 20,000) [19], the excess number of deaths in IA steroid users was higher, suggesting more deaths by other causes than cardiovascular events in the comparison group vs. the exposed group. These excess deaths by other causes among IA steroid users lead to an overestimated cardioprotective effect of chondroitin in this analysis. Thus, we must assume that some residual confounding by patient frailty may also play a role in the results of our primary outcome. Patient frailty is a covariate that is difficult to fully approximate in Swiss claims databases.

The effect estimates in the as-started analyses did not meaningfully change compared to the main primary analysis, and the effect was also strongest within the first year of follow-up. Interestingly, patients who claimed only one chondroitin treatment course showed a stronger effect estimate (mHR 0.80; 95% CI 0.72–0.88) during three years of follow-up after a 180-day run-in period, i.e. after they had stopped taking chondroitin. The potential mode of action of how chondroitin might lead to a cardioprotective effect is unknown, but it is unlikely that one single claim of chondroitin would causally lead to a lasting cardioprotective effect beyond the end of this treatment course. Thus, this analysis is a further indicator for the presence of some potential residual confounding.

The post-hoc analysis applying PS fine stratification was consistent with our primary outcome analysis with a comparable HR (0.83 vs. 0.81) while retaining >99.9% of patients in both groups (compared to only 40.7% of chondroitin users after PS matching) suggesting that generalizability of our primary analysis is given.

We used a representative and large Swiss healthcare claims database and applied rigorous methodology to control for confounding. However, some limitations need to be considered. First, other than prior studies, we did not include glucosamine as exposure in our analysis because in Switzerland, glucosamine is not reimbursed by health insurance. Glucosamine would have been an interesting exposure to evaluate, given that prior studies observed a risk reduction for ischemic stroke, but not for myocardial infarction [2,3]. Second, we had no information on lifestyle factors, such as smoking, alcohol consumption, physical activity, or BMI. These factors are risk factors for cardiovascular events, and high BMI and low physical activity are associated with an increased risk of OA (or a consequence thereof) [20]. While our negative control analysis for hernia suggests that BMI is not a relevant confounder in our study, we cannot rule out potential confounding by other life-style factors. We can also not rule out healthy-user bias given that chondroitin is a drug without an immediately perceived benefit. Third, we did not have information about medication adherence and therefore do not know if patients took the chondroitin they claimed. Furthermore, median follow-up of chondroitin use was 180 days, which corresponds to one single chondroitin claim. Thus, more than 50% of chondroitin users had only one claim for chondroitin, despite the chronic nature of OA. Thus, our results only reflect short-term use of chondroitin, and we cannot speculate on a long-term effect of chondroitin on the cardiovascular risk. Fourth, while additional comparison groups would be desirable to test robustness of our results, all considered additional comparison groups were either not available or would have introduced a greater risk of confounding; topical nonsteroidal anti-inflammatory drugs are often purchased over the counter and like non-NSAID analgesics have a broader range of non-OA indications. Intraarticular hyaluronic acid injections are not reimbursed by health insurance in Switzerland and non-users are unlikely an exchangeable comparison group. Finally, while the effect mechanism of chondroitin on the cartilage is known [1], there is no evidence on a potential effect mechanism that would explain a potential cardioprotective effect. This complicates the interpretation of the observed association in terms of how, when, and how long for a potential causal cardioprotective effect of chondroitin would be present. Despite these limitations, this is, to our knowledge, the first study using active comparison and applying PS matching to control for confounding to assess the association between chondroitin and the risk of acute thromboembolic cardiovascular events.

## 5 Conclusion

In our PS-matched cohort study comparing new users of chondroitin to new users of IA steroids, we observed strong mHRs in association with new chondroitin use. These results may indicate a potential cardioprotective effect of chondroitin, but residual confounding may partly explain the decrease in effect estimates. Further research is needed to evaluate whether this intriguing observed association may be causal or not.

## Author contributions

T. Zappalà: First author, contribution to the study design, acquisition of data, performing analyses, interpreting results, and drafting the work, as well as finally approving the version to be published. T. Burkard: Contribution to the design of the work, performing analyses, critically reviewing for important intellectual content, and finally approving the version to be published. C.A. Huber: Contribution to the design of the work, acquisition of data, reviewing the work critically for important intellectual content, and finally approving the version to be published. J. Spoendlin: Contribution to the study design, interpreting results, drafting the work and reviewing it critically, and finally approving the version to be published. C. R. Meier: Corresponding author, contribution to the design of the work, reviewing the work critically for important intellectual content, and finally approving the version to be published.

## Funding information

This study was funded by an unconditional research grant by IBSA (Institut Biochimique SA).

## Conflict of interest

The University of Basel (C.R.Meier and J.Spoendlin) received an unconditional research grant by IBSA (Institut Biochimique SA) to conduct this study. The responsibility for composing the study design and interpreting results was with the University of Basel alone.

## References

[1] HCI Solutions AG (2024). https://www.swissmedicinfo.ch/.

[2] Mazzucchelli R., Rodríguez-Martín S., García-Vadillo A., Gil M., Rodríguez-Miguel A., Barreira-Hernández D. (2021). Risk of acute myocardial infarction among new users of chondroitin sulfate: a nested case-control study. PLoS One.

[3] Mazzucchelli R., Rodríguez-Martín S., Crespí-Villarías N., García-Vadillo A., Gil M., Izquierdo-Esteban L. (2022). Risk of ischaemic stroke among new users of glucosamine and chondroitin sulphate: a nested case–control study. Ther. Adv. Musculoskelet. Dis..

[4] Schneeweiss S., Patrick A.R., Stürmer T., Brookhart M.A., Avorn J., Maclure M. (2007). Increasing levels of restriction in pharmacoepidemiologic database studies of elderly and comparison with randomized trial results. Med. Care.

[5] Hawker G.A., Croxford R., Bierman A.S., Harvey P.J., Ravi B., Stanaitis I. (2014). All-cause mortality and serious cardiovascular events in people with hip and knee osteoarthritis: a population based cohort study. PLoS One.

[6] Barbour K.E., Lui L.-Y., Nevitt M.C., Murphy L.B., Helmick C.G., Theis K.A. (2015). Hip osteoarthritis and the risk of all-cause and disease-specific mortality in older women: a population-based cohort study. Arthritis Rheumatol..

[7] Hochberg M.C. (2008). Mortality in osteoarthritis. Clin. Exp. Rheumatol..

[8] Wang Z., Kang C., Xu P., Zhang S., Song J.H., Wang D. (2022). Osteoarthritis and cardiovascular disease: a mendelian randomization study. Front. Cardiovasc. Med..

[9] Kendzerska T., Jüni P., King L.K., Croxford R., Stanaitis I., Hawker G.A. (2017). The longitudinal relationship between hand, hip and knee osteoarthritis and cardiovascular events: a population-based cohort study. Osteoarthr. Cartil..

[10] Kendzerska T., King L.K., Lipscombe L., Croxford R., Stanaitis I., Hawker G.A. (2018). The impact of hip and knee osteoarthritis on the subsequent risk of incident diabetes: a population-based cohort study. Diabetologia.

[11] Schur N., Twerenbold S., Rothweiler L.J., Blankart K., Fischer R., Stöckle M. (2023).

[12] Dietz U.A., Kudsi O.Y., Gokcal F., Bou-Ayash N., Pfefferkorn U., Rudofsky G. (2021). Excess body weight and abdominal hernia. Visc. Med..

[13] Rubin D.B. (1997). Estimating causal effects from large data sets using propensity scores. Ann. Intern. Med..

[14] Desai R.J., Rothman K.J., Bateman B.T., Hernandez-Diaz S., Huybrechts K.F. (2017). A propensity-score-based fine stratification approach for confounding adjustment when exposure is infrequent. Epidemiology.

[15] Bas H. (2021). Arthrosetherapie - was sagen die internationalen guidelines. Congr. Rheumatol..

[16] Zhang Y., Jordan J.M. (2010). Epidemiology of osteoarthritis. Clin. Geriatr. Med..

[17] Swiss Health Observatory (2025). Swiss health observatory. Akuter Myokardinfarkt.

[18] Swiss Health Observatory (2025). Swiss health observatory. Stroke.

[19] Bundesamt für Gesundheit (BAG). Herz-Kreislauf-Erkrankungen 2018. https://www.bag.admin.ch/bag/de/home/krankheiten/krankheiten-im-ueberblick/herz-kreislauf-erkrankungen.html (accessed January 30, 2025).

[20] Palazzo C., Nguyen C., Lefevre-Colau M.M., Rannou F., Poiraudeau S. (2016). Risk factors and burden of osteoarthritis. Ann. Phys. Rehabil. Med..

