# Supplementary material for: The risk of acute thromboembolic cardiovascular events in association with use of chondroitin sulphate: A comparative, propensity score-matched cohort study using Swiss healthcare claims data
Source: Osteoarthr Cartil Open. 2026 Apr 2;8(2):100791. doi: 10.1016/j.ocarto.2026.100791 (PMC13092033; doi:10.1016/j.ocarto.2026.100791)
Supplement: Multimedia component 1 [file mmc1.docx]

**Supplementary material**

# **Supplementary methods**

## *M1: Exposure definitions (Table S1)*

***Table S1: Pharmacodes and TARMED codes for the definition of IA steroid exposure***

| **Codes** | **Specifications** |
| --- | --- |
| *Pharmacodes* | |
| 66654 | KENACORT A 10 Inj Susp 50 mg/5ml |
| 654109 | KENACORT A 10 Inj Susp 10 mg/ml, Ampulle 1 ml |
| 1547858 | KENACORT A 10 Inj Susp 10 mg/ml, 5 Ampullen x 1 ml |
| 66683 | KENACORT A 40 Inj Susp 40 mg/ml, Ampulle 1 ml |
| 912830 | KENACORT A 40 Inj Susp 40 mg/ml, Spritzampulle 1 ml |
| 1528453 | KENACORT A 40 Inj Susp 40 mg/ml, 5 Ampullen x 1 ml |
| 574391 | KENACORT A 40 Inj Susp 200 mg/5ml |
| 5612736 | KENACORT A Solubile Inj Lös 40 mg/1ml |
| *TARMED* | |
| 24.0130 | Gelenkpunktion (inkl. Ganglion, Gelenkzyste), Schulter, Ellbogen, Knie, oberes Sprunggelenk OSG |

## *M2: Outcome definitions (Tables S2 and S3)*

***Table S2: SwissDRG codes for primary outcome of interest***

| **Myocardial infarction** | |
| --- | --- |
| F41A | Invasive kardiologische Diagnostik bei akutem Myokardinfarkt mit äusserst schweren CC |
| F41B | Invasive kardiologische Diagnostik bei akutem Myokardinfarkt |
| F60A | Akuter Myokardinfarkt ohne invasive kardiologische Diagnostik mit äusserst schweren CC, mehr als ein Belegungstag |
| F60B | Akuter Myokardinfarkt ohne invasive kardiologische Diagnostik, mehr als ein Belegungstag |
| **Ischemic Stroke** | |
| B70A | Apoplexie mit neurologischer Komplexbehandlung des akuten Schlaganfalls > 72 Std., mit komplizierender Diagnose od. schw. motor. Funktionsstörung |
| B70B | Apoplexie mit neurologischer Komplexbehandlung des akuten Schlaganfalls > 72 Std. od. neurolog. Komplexdiagnostik |
| B70C | Apoplexie mit neurologischer Komplexbehandlung des akuten Schlaganfalls < 73 Std., mit komplizierender Diagnose oder Thrombolyse oder schw. motor. Funktionsstörung |
| B70D | Apoplexie mit neurologischer Komplexbehandlung des akuten Schlaganfalls < 73 Std., oder mit anderer neurolog. Komplexbehandlung > 72 Std. |
| B70E | Apoplexie mit anderer neurologischer Komplexbehandlung des akuten Schlaganfalls < 73 Std. od. neurolog. Komplexdiagnostik |
| B70F | Apoplexie, mehr als ein Belegungstag oder Thrombolyse mit komplizierender Diagnose oder schw. motor. Funktionsstörung |
| B70G | Apoplexie, mehr als ein Belegungstag |
| B70H | Apoplexie, mehr als ein Belegungstag oder Thrombolyse, verstorben < 5 Tage nach Aufnahme, mit neurologischer Komplexbehandlung des akuten Schlaganfalls oder anderer neurologischer Komplexbehandlung |
| B70I | Apoplexie, mehr als ein Belegungstag oder Thrombolyse, verstorben < 5 Tage nach Aufnahme |
| B70J | Apoplexie mit neurologischer Komplexbehandlung des akuten Schlaganfalls > 23 Std. bis < 48 Std., ein Belegungstag |
| B70K | Apoplexie mit neurologischer Komplexbehandlung des akuten Schlaganfalls < 24 Std., ein Belegungstag |
| B39A | Neurologische Komplexbehandlung des akuten Schlaganfalls mit bestimmter Prozedur od. Apoplex /TIA mit Herzschrittmacher, mehr als 72 Stunden mit komplexem Eingriff oder mit komplizierender Prozedur |
| B39B | Neurologische Komplexbehandlung des akuten Schlaganfalls mit bestimmter Prozedur, bis 72 Stunden mit komplexem Eingriff, oder mehr als 72 Stunden |
| B39C | Neurologische Komplexbehandlung des akuten Schlaganfalls mit bestimmter Prozedur od. Apoplex/TIA mit ASD Verschluss |
| A95B | Geriatrische Akutrehabilitation ab 14 Behandlungstagen od. neurologische Komplexbehandlung des akuten Schlaganfalls, mit bestimmter OR Prozedur od. Intensivmedizinische Komplexbehandlung > 184 Aufwandspunkte |
| A95C | Geriatrische Akutrehabilitation ab 14 Behandlungstagen oder neurologische Komplexbehandlung des akuten Schlaganfalls, mit schwerer motorischer Funktionseinschränkung oder bestimmtem Eingriff |
| A95D | Geriatrische Akutrehabilitation ab 14 Behandlungstagen oder neurologische Komplexbehandlung des akuten Schlaganfalls |
| B42A | Frührehabilitation bei Krankheiten und Störungen des Nervensystems bis 27 Tage, mit neurologischer Komplexbehandlung des akuten Schlaganfalls |
| B44A | Geriatrische frührehabilitative Komplexbehandlung bei Krankheiten und Störungen des Nervensystems mit schwerer motorischer Funktionseinschränkung, mit neurologischer Komplexbehandlung des akuten Schlaganfalls |
| B44C | Geriatrische frührehabilitative Komplexbehandlung bei Krankheiten und Störungen des Nervensystems ohne schwere motorische Funktionseinschränkung, mit neurologischer Komplexbehandlung des akuten Schlaganfalls |
| **Transient ischemic attack (TIA)** | |
| B69A | Transitorische ischämische Attacke (TIA) und extrakranielle Gefässverschlüsse mit neurologischer Komplexbehandlung des akuten Schlaganfalls > 72 Stunden |
| B69B | Transitorische ischämische Attacke (TIA) und extrakranielle Gefässverschlüsse mit neurologischer Komplexbehandlung des akuten Schlaganfalls < 73 Stunden |
| B69C | Transitorische ischämische Attacke (TIA) und extrakranielle Gefässverschlüsse mit anderer neurologischer Komplexbehandlung des akuten Schlaganfalls od. neurolog. Komplexdiagnostik |
| B69D | Transitorische ischämische Attacke (TIA) und extrakranielle Gefässverschlüsse |
| B69E | Transitorische ischämische Attacke (TIA) und extrakranielle Gefässverschlüsse ohne neurologische Komplexbehandlung des akuten Schlaganfalls, ohne äusserst schwere CC |

***Table S3: SwissDRG and ATC codes for the negative control outcome***

| **Hernia surgery (SwissDRG)** | |
| --- | --- |
| G09A | Eingriffe bei Hernien mit äusserst schweren CC, mehr als ein Belegungstag |
| G09B | Beidseitige Eingriffe bei Leisten- und Schenkelhernien mit Narbenhernie, mehr als ein Belegungstag |
| G09C | Beidseitige Eingriffe bei Leisten- und Schenkelhernien, mehr als ein Belegungstag |
| G09D | Eingriffe bei Narbenhernien mit Transplantat, mehr als ein Belegungstag |
| G09E | Eingriffe bei Leisten- und Schenkelhernien mit Transplantat, mehr als ein Belegungstag |
| G09F | Eingriffe bei Narbenhernien, mehr als ein Belegungstag |
| G09G | Eingriffe bei Leisten- und Schenkelhernien, mehr als ein Belegungstag |
| G09H | Eingriffe bei Hernien, ein Belegungstag |
| G09Z | Beidseitige Eingriffe bei Leisten- und Schenkelhernien, Alter > 55 Jahre oder komplexe Herniotomien |
| G24Z | Eingriffe bei Bauchwandhernien, Nabelhernien und anderen Hernien, Alter > 0 Jahre oder beidseitige Eingriffe bei Leisten- und Schenkelhernien, Alter > 0 Jahre und < 56 Jahre oder Eingriffe bei Leisten- und Schenkelhernien, Alter > 55 Jahre |

# **Supplementary results**

***Table S4: Distribution of baseline covariates of chondroitin users and IA steroid users in the sensitivity analysis before and after PS fine stratification***

|  | Before PS fine stratification | | | PS fine stratification | | |
| --- | --- | --- | --- | --- | --- | --- |
|  | **Chondroitin**  **(N=90 060)** | **IA steroids**  **(N=37 726)** | **SMD** | **Chondroitin**  **(N=90 058)** | **IA steroids**  **(N=37 721)** | **SMD** |
| Mean age [years] (SD) | 63.7 (12.1) | 65.9 (13.1) | -0.173 | 63.7 (12.1) | 63.7 (12.2) | 0.000 |
| Male | 31 444 (34.9%) | 15 380 (40.8%) | -0.121 | 31 444 (34.9%) | 13 117 (34.8%) | 0.003 |
| Mean number of claims (of medications listed below) 365d before CED (SD) | 8.3 (7.7) | 10.7 (9.4) | -0.278 | 8.3 (7.5) | 8.4 (7.9) | -0.020 |
| Medications 365d prior CED: | | | | | | |
| Reflux medication (ATC A02) | 40 288 (44.7%) | 20 309 (53.8%) | -0.183 | 40 287 (44.7%) | 16 765 (44.4%) | 0.006 |
| Antidiabetics (ATC A10) | 8 132 (9.0%) | 4 177 (11.1%) | -0.068 | 8 131 (9.0%) | 3 415 (9.1%) | -0.001 |
| Anticoagulants (ATC B01AA / B01AB / B01AF) | 11 463 (12.7%) | 6 098 (16.2%) | -0.098 | 11 463 (12.7%) | 4 988 (13.2%) | -0.015 |
| Blood substitutes (ATC B05) | 21 287 (23.6%) | 10 963 (29.1%) | -0.123 | 21 286 (23.6%) | 8 903 (23.6%) | 0.001 |
| Cardiac therapy (ATC C01) | 8 727 (9.7%) | 4 473 (11.9%) | -0.070 | 8 727 (9.7%) | 3 660 (9.7%) | 0.000 |
| Diuretics (ATC C03) | 7 574 (8.4%) | 4 770 (12.6%) | -0.138 | 7 573 (8.4%) | 3 220 (8.5%) | -0.005 |
| Vasodilators (ATC C04) | 11 611 (12.9%) | 4 913 (13.0%) | 0.004 | 11 611 (12.9%) | 4 937 (13.1%) | -0.006 |
| Betablockers (ATC C07) | 16 123 (17.9%) | 8 199 (21.7%) | -0.096 | 16 122 (17.9%) | 6 773 (18.0%) | 0.001 |
| Calcium-channel blockers (ATC C08) | 8 788 (9.8%) | 4 716 (12.5%) | -0.087 | 8 787 (9.8%) | 3 724 (9.9%) | 0.004 |
| RAAS-blockers (ATC C09) | 29 633 (32.9%) | 14 449 (38.3%) | -0.113 | 29 632 (32.9%) | 12 342 (32.7%) | 0.004 |
| Lipid-modifiers (ATC C10) | 21 294 (23.6%) | 10 156 (26.9%) | -0.075 | 21 293 (23.6%) | 8 882 (23.5%) | 0.002 |
| Secondary Antihypertensives (ATC C02) | 625 (0.7%) | 362 (1.0%) | -0.029 | 625 (0.7%) | 271 (0.7%) | 0.003 |
| Thyroid therapy (ATC H03) | 7 597 (8.4%) | 3 195 (8.5%) | 0.001 | 7 597 (8.4%) | 3 208 (8.5%) | 0.002 |
| Anti-inflammatory and antirheumatic products (ATC M01A, excl. chondroitin sulphate) | 58 420 (64.9%) | 26 907 (71.3%) | -0.139 | 58 419 (64.9%) | 24 399 (64.7%) | 0.004 |
| Topical pain medication (ATC M02) | 35 196 (39.1%) | 14 999 (39.8%) | -0.014 | 35 194 (39.1%) | 14 759 (39.1%) | 0.001 |
| Opioids (ATC N02A) | 13 405 (14.9%) | 8 654 (22.9%) | -0.207 | 13 404 (14.9%) | 5 700 (15.1%) | -0.006 |
| Analgesics (ATC N02B) | 41 316 (45.9%) | 20 136 (53.4%) | -0.150 | 41 314 (45.9%) | 17 302 (45.9%) | 0.000 |
| Psycholeptics (ATC N05) | 23 448 (26.0%) | 10 876 (28.8%) | -0.063 | 23 446 (26.0%) | 9 910 (26.3%) | -0.005 |
| Bone drugs (ATC M05B) | 3 977 (4.4%) | 2 208 (5.9%) | -0.065 | 3 977 (4.4%) | 1 643 (4.4%) | 0.003 |
| Procedures 365d before CED | | | | | | |
| Outpatient arthroscopy | 1 183 (1.3%) | 617 (1.6%) | -0.027 | 1 183 (1.3%) | 525 (1.4%) | -0.007 |
| MRI | 16 885 (18.7%) | 10 730 (28.4%) | -0.230 | 16 885 (18.7%) | 6 950 (18.4%) | 0.008 |
| X-ray | 30 897 (34.3%) | 12 587 (33.4%) | 0.020 | 30 897 (34.3%) | 13 272 (35.2%) | -0.018 |
| Arthroplasty (any time before CED) | 849 (0.9%) | 776 (2.1%) | -0.092 | 849 (0.9%) | 387 (1.0%) | -0.008 |
